# Intraocular gases and climate change: a call for sustainable vitreoretinal surgery

**DOI:** 10.3389/fpubh.2025.1706042

**Published:** 2025-12-02

**Authors:** Joseph Paolo Y. Silva, Recivall P. Salongcay

**Affiliations:** 1 Xavier School, San Juan, Philippines; 2 Eye and Vision Institute, The Medical City, Pasig, Philippines

**Keywords:** healthcare sustainability, sulfur hexafluoride, vitreoretinal surgery, sustainable surgical care, carbon footprint

## Abstract

Perfluoropropane (C₃F₈) and sulfur hexafluoride (SF₆) are established agents in vitreoretinal surgery. Their tamponade properties support anatomic success, but both gases have very high global warming potentials and extremely long atmospheric lifetimes. Given the health sector’s considerable share of global greenhouse emissions, emissions attributable to intraocular gases constitute a discrete, measurable, and modifiable component of surgical practice. This Perspective synthesizes published evidence on the climate impact of C₃F₈ and SF₆ in routine vitreoretinal care and outlines actions at three levels. First, clinical practice: standardize low-concentration mixtures, match prepared volume to need, improve decanting technique, and consider air tamponade in appropriate indications. Second, implementation systems: training, checklists, and simple process metrics (prepared-to-injected ratios; concentration adherence) to reduce variation and waste. Third, institutional and policy measures: procurement criteria that favor lower GWP options and right sized packaging, guideline updates, audit indicators, and product level carbon disclosure. These steps do not introduce new clinical risk when applied with standard safeguards and may yield cost savings by reducing gas consumption. Because fluorinated intraocular gases are potent, long lived, and tied to modifiable routines, targeted measures in this niche can produce outsized gains for planetary health relative to effort.

## Introduction

Fluorinated intraocular gases, perfluoropropane (C₃F₈) and sulfur hexafluoride (SF₆), are essential in rhegmatogenous retinal detachment repair, vitreoretinal surgical procedures and related indications (1). Their durability in the vitreous cavity provides stable tamponade that supports reattachment and postoperative healing. These same physicochemical features, however, confer a high climate impact: on a 100 year horizon, the global-warming potential (GWP₁₀₀) of C₃F₈ is roughly 7,000 and that of SF₆ approximately 23,500 relative to carbon dioxide; both persist in the atmosphere for thousands of years once released (2). In health systems where operating rooms are among the most energy and material intensive environments (3), these properties justify targeted attention to how intraocular gases are prepared and used.

Although emissions from a single retinal case may seem modest, regional atmospheric data show that sulfur hexafluoride (SF₆) emissions are increasing significantly (4). A typical vitreoretinal case using 20% SF₆ involves approximately 120 mL of pure gas, corresponding to about 2.8 kg CO₂-equivalent. For an institution performing 500 retinal surgeries annually, this represents roughly 1.4 tonnes CO₂-equivalent each year, comparable to the emissions produced by burning 1,700 pounds of coal. In ophthalmology, national professional bodies now frame mitigation as part of clinical quality, with recommendations for daily practice and procurement reform (5, 6). These developments, together with the long atmospheric persistence and very high global-warming potentials of C₃F₈ and SF₆ (2), justify specific targeted measures in vitreoretinal surgery. This perspective situates intraocular gas use within planetary health, summarizes the evidence for mitigation, and proposes clinical and system actions that maintain outcomes while reducing emissions.

### The environmental burden of intraocular gases

The climate impact of intraocular gases reflects both gas properties and handling practices. At the property level, SF₆ exhibits a higher GWP₁₀₀ than C₃F₈ and is exceptionally stable in the atmosphere (2). Atomistic simulations indicate that thermal degradation pathways for SF₆ occur only at very high temperatures, helping to explain the centuries-long persistence of small releases (7). At the practice level, preparation and delivery systems drive much of the footprint: large cylinders and over-preparation increase loss, whereas smaller canisters and standardized concentrations reduce it (8). A five-year retrospective analysis at a tertiary eye hospital quantified volumes of SF₆, C₂F₆, and C₃F₈ across vitreoretinal procedures and converted usage to CO₂-equivalent, showing that case mix, gas choice, and dilution protocols are the principal drivers of the gas-related footprint in day-to-day practice (9). National activity-based estimates suggest that, when aggregated, fluorinated intraocular gases contributed approximately 201 tonnes CO₂-equivalent over a decade (~18 t per year), a small share of health-sector emissions; nonetheless, these emissions are avoidable and long-lived and therefore remain appropriate targets for reduction (10).

The upstream supply chain also matters. Packaging sizes, canister design, and mixing/decanting accessories influence how much gas is prepared versus injected. Wastage becomes routine when packaging promotes over-preparation. Furthermore, because these agents are long-lived, releases today persist in the atmosphere for centuries to millennia, so reductions achieved now confer sustained climate benefit. The environmental impact of intraocular gas use is well defined, involving only two gases with a limited set of standardized preparation steps, making it ideal for targeted intervention.

### Strategies for emissions reduction associated with intraocular gas use

#### Standardized low-concentration mixtures

Lower concentration mixtures (e.g., 12–14% C₃F₈) maintain anatomical success while substantially reducing gas related emissions; published estimates suggest reductions of up to 80% relative to higher concentration approaches (8). Standardization converts knowledge to practice: define a default concentration range in preference cards and order sets; post dilution tables at preparation benches; and verify concentration before injection (8, 11). Prefilled options or kits, if feasible, can further reduce variation.

Gas-type selection also affects emissions. Because SF₆ has a higher GWP₁₀₀ than C₃F₈, avoiding SF₆ or using lower-fraction mixtures provides additional benefit where tamponade physics permit. Standardized mixtures and prefilled options curb handling loss (8), while air tamponade eliminates fluorinated-gas emissions entirely in suitable cases (11).

A recent commentary proposes a stepwise hierarchy for reducing fluorinated gas use: default to air if clinically appropriate; when a gas is needed, favor lower-concentration mixtures and agents with lower global-warming potential than SF₆; tighten decanting and packaging to curb waste; and track service-level usage metrics (12). Within this hierarchy, dilute low fraction C₂F₆ (8%) has been proposed as an alternative to SF₆ (20%) in selected indications, with substitution guided by tamponade requirements and local outcomes monitoring (12).

#### Volume preparation matched to surgical need

Prepared syringe volume should reflect the expected intraoperative requirement based on procedure type and ocular measurements, rather than fixed “buffer” overfill. The healthcare team can incorporate volume targets into pre-case briefings and record both prepared and injected volumes post-case.

#### Low-loss gas decanting technique

Losses during transfer can be minimized with slow, controlled decanting using closed or semi-closed systems and careful monitoring of plunger motion. Short, laminated step guides at the preparation station reduce variability and minimize errors. These steps require training and consistency rather than new equipment.

#### Air tamponade in selected indications

For selected indications, air tamponade can yield clinical outcomes comparable to fluorinated gases while eliminating fluorinated emissions (11). Indications should be defined, and early adoption monitored with routine anatomical and visual outcomes. More importantly, forthcoming studies should report environmental endpoints alongside clinical results to support balanced decisions.

### Implementation systems: training, checklists, and feedback

Durable change requires making efficient practice the default. Teach accurate dilution, volume matching, and low-loss decanting in specialty training and continuing education, with simulation when possible. Put these expectations into checklists and preference cards that specify target concentration and prepared volume, and add a brief “no excess preparation” reminder to pre-case briefings. At the preparation stations, post dilution tables, set default orders to low-concentration mixtures, favor small-volume canisters when available, and document prepared and injected volumes. Review charts of concentration adherence, prepared-to-injected ratios, and tamponade choice by indication alongside clinical outcomes (reattachment, complications and return to the operating room) at regular service meetings so conservation is audited with the same cadence as quality and safety (5, 6).

### Institutional levers: procurement and supply chain

Procurement can reduce loss before the operating room. Hospitals can request right sized canisters, prefilled low-concentration options, and accessories that support closed or semi-closed transfer. Environmental product declarations and life cycle assessments should be requested for gas products and accessories so that purchasing decisions incorporate carbon emissions information alongside price and performance. Contracts can include sustainability clauses that favor suppliers offering lower GWP options and staff training.

### Policy and professional standards

Clinical guidelines can specify low-concentration mixtures if suitable, provide practical decanting guidance, and list indications for air tamponade. Accreditation bodies can include a small set of environmental indicators—percentage of cases within target concentration, median prepared-to-injected ratios—so that sustainability is handled within quality and safety processes rather than as an optional add on. Regulators can encourage product level carbon disclosure and inclusion of environmental endpoints in post market studies. Such measures create consistent expectations across institutions and reduce reliance on individuals.

The policy context outside medicine is evolving as regulators respond to rising SF₆ emissions (4). Although ophthalmology contributes a small share at the over-all scale, aligning procurement with lower-GWP choices and right-sized packaging reduces waste and mitigates exposure to future supply constraints.

These ophthalmology-specific steps align with cross-disciplinary guidance on medical greenhouse gases, which emphasizes avoiding high-GWP agents where viable alternatives exist and improving product-level emissions disclosure (13).

### Equity and global context

Populations most exposed to climate hazards often have the least access to specialist eye care and the least capacity to adapt. Approaches that reduce gas usage per case—lower concentration mixtures, matched volume, careful decanting, air when appropriate—can lower consumables spending, freeing resources for access and follow up. Training resources should be adaptable for varied settings: short videos, pictorial step guides, and simple checklists that can be translated and applied without specialized equipment. Partnerships across income settings can share tools and recommendations while respecting local constraints. Professional statements increasingly call for pragmatic, implementable steps of this kind in eye care (5, 6).

### Economic considerations

Many measures reduce cost as they reduce carbon. Avoiding routine over preparation, matching volume to need, and defaulting to lower concentration mixtures use less gas per successful case. If air tamponade is appropriate, the gas cost is removed and fluorinated emissions are avoided. Procurement reforms—smaller canisters; prefilled options—can cut waste and the labor required for rework. Budget impact depends on local pricing and contracts, but the direction is consistent: practices that reduce emissions generally waste fewer materials and staff time.

### Quantifying metrics for improvement

For each case, record prepared gas volume and concentration, injected volume, and tamponade type (air vs. fluorinated gas). Use standard global-warming potentials to convert gas volume and concentration into CO₂-equivalent per case. From these data, report service indicators at regular intervals: the prepared-to-injected ratio (lower is better) and the percentage of cases within the target concentration. If helpful, add a brief summary of median CO₂-equivalent per case by indication and the proportion of eligible cases managed with air. Present these alongside clinical outcomes (reattachment rates, complications) so conservation is reviewed with the same cadence and rigor as other quality measures, consistent with recommendations to integrate sustainability into routine audit (5) (see Table 1).

**Table 1:** Estimated carbon footprint reduction achievable through volume-matched preparation and checklist implementation for intraocular gas use in pars plana vitrectomy.

Practice element	Conventional approach (20% SF₆, over-prepared)	Improved approach (20% SF₆, volume-matched + checklist)	Reduction
Pure SF₆ used (mL)	120	6	95%
Prepared-to-injected ratio	10: 1	5: 1	50%
CO₂-equivalent per case (kg)	2.8	0.14	≈ 20 × lower
Annual emissions (500 cases)	1.4 tonnes	0.07 tonnes	−1.33 tonnes CO₂-eq

SF₆, sulfur hexafluoride; CO₂-eq, carbon dioxide equivalent; t, tonne (1 tonne = 1,000 kg).

The table compares conventional and improved preparation approaches for a typical rhegmatogenous retinal detachment case using 20% sulfur hexafluoride (SF₆). Under standard practice, 120 mL of pure SF₆ is used to prepare a 60 mL syringe, though only 12 mL is injected with a prepared-to-injected ratio of 10:1. Implementing standardized 20% mixtures, matching prepared volume to surgical need (e.g., 30 mL prepared, 6 mL injected), and introducing a “no-excess” checklist reduces per-case emissions from 2.8 kg CO₂-equivalent to 0.14 kg CO₂-equivalent—approximately a twenty-fold decrease. Extrapolated over 500 annual cases, this corresponds to a reduction of about 1.33 tonnes CO₂-equivalent per year.

### Research priorities

Several areas merit prospective evaluation. First, outcomes with low-concentration mixtures across indication subgroups with standardized anatomical and visual endpoints. Second, comparative studies of air versus gas tamponade that include environmental endpoints and patient reported outcomes. Third, assessments of closed system transfer and prefilled options quantify time, waste, cost, and emissions. Fourth, implementation research to define education and feedback strategies that sustain change across diverse settings. Journals and funders can support this by requesting environmental reporting in tamponade comparisons and by providing tools for case level carbon estimation.

### Limitations

Environmental figures cited here derive from clinical cohorts and national activity-based estimates that differ in scope and assumptions; absolute values are context-dependent. Mechanistic work indicates that SF₆ is chemically resilient, with degradation pathways activated only at very high temperatures, therefore, perioperative releases will therefore persist in the atmosphere for thousands of years. These considerations argue for transparency about system boundaries and assumptions in any local carbon accounting.

## Conclusion

Intraocular gases deliver clear clinical benefit but carry a disproportionate climate cost because of high GWP and long persistence (2). The practical response is to minimize use and loss without compromising outcomes: default to low-concentration mixtures, right-size prepared volumes, use closed or semi-closed transfer, choose air for suitable indications, and favor packaging and systems that reduce waste. Recent evidence suggests that although retinal surgery make up a small share of gas emissions, the impact and atmospheric persistence of rising SF₆ emissions, supports tighter control wherever this gas is used. Professional guidance supports integrating simple metrics and procurement choices into routine governance. Mechanistic studies emphasize that avoided releases today prevent centuries-long atmospheric persistence. These steps reduce a defined, avoidable source of emissions in retinal care while maintaining visual outcomes for patients.

## Data Availability

The original contributions presented in the study are included in the article/supplementary material, further inquiries can be directed to the corresponding author.
